# Synergistic Silk Fibroin/Cellulose Inverse Opals as Flexible Colorimetric Sensors for Multiphase Water and Organic Alcohol Recognition

**DOI:** 10.3390/s26123875

**Published:** 2026-06-18

**Authors:** Jiong Guo, Yue Wang, Dan Wu, Lili Qiu, Zhibin Xu, Junming Geng, Yifei Wang, Zihui Meng

**Affiliations:** 1 School of Chemistry and Chemical Engineering, Beijing Institute of Technology, Beijing 100081, China; guojiong@bit.edu.cn (J.G.);; 2 Yangtze Delta Region Academy, Beijing Institute of Technology, Jiaxing 314000, China

**Keywords:** silk fibroin, inverse opals, colorimetric moisture sensing

## Abstract

A silk fibroin/cellulose inverse-opal photonic crystal composite with robust mechanical properties was fabricated by blending a silk fibroin solution with methylcellulose, utilizing a 3D poly(methyl methacrylate) (PMMA) photonic crystal array as a template, via sequential infiltration, curing, and etching processes. Leveraging the intrinsic water sensitivity of both silk fibroin and methylcellulose, the resulting composite exhibits exceptional moisture-sensing capabilities across gaseous, liquid, and solid phases. Specifically, for atmospheric humidity, the film delivers a distinct optical response to a relative humidity variation in merely 5%. In liquid systems, owing to the material’s excellent affinity for low-polarity organic solvents and the disruptive effect of highly polar solvents (e.g., water) on the photonic periodic structure, the structural color of the film can sensitively report trace water contents down to 0.025%. Furthermore, in solid matrices, the composite enables the precise detection of not only free water but also water of crystallization.

## 1. Introduction

In nature, the vibrant and iridescent coloration observed in various organisms, such as Morpho butterfly wings, beetle exoskeletons, and peacock feathers, originates from highly ordered micro- and nanostructures known as photonic crystals [1]. Unlike traditional chemical pigments and dyes, which are inevitably susceptible to photobleaching and environmental degradation, these structural colors are generated by the physical interaction of light—such as interference, diffraction, and scattering—with periodic dielectric structures [2]. Consequently, bio-inspired structural colors exhibit exceptional long-term optical stability, high brightness, and tunable coloration. Furthermore, when constructed from natural biomaterials, these photonic crystals offer unparalleled advantages, including inherent biocompatibility, biodegradability, and eco-friendliness [3]. These compelling attributes render bio-based photonic materials highly attractive candidates for replacing toxic synthetic pigments and developing sustainable, next-generation optical sensors and smart responsive platforms.

The accurate detection and continuous monitoring of moisture content are of paramount importance across diverse fields, ranging from agricultural grain storage and food preservation to industrial chemical processing and environmental monitoring. Conventional moisture detection techniques, such as capacitance-based sensors, resistive hygrometers, and thermogravimetric analysis (TGA), are widely employed due to their quantitative capabilities [4,5]. However, these traditional methods often suffer from inherent limitations, including the reliance on complex and bulky instrumentation, high operational costs, the need for a continuous power supply, and crucially, a lack of direct, naked-eye visual feedback. To overcome these bottlenecks, there is a burgeoning demand for portable, power-free, and colorimetric moisture sensors. In this context, silk fibroin (SF), a natural structural protein extracted from Bombyx mori cocoons, has emerged as a highly promising building block [6]. SF not only possesses excellent mechanical robustness, optical transparency, and biocompatibility, but also exhibits profound intrinsic hygroscopicity due to its abundant hydrophilic amino acid residues, making it an ideal matrix for humidity-responsive optical materials [7].

In recent years, the integration of silk fibroin with photonic crystal architectures [8,9] has garnered significant research interest, particularly for the development of advanced stimuli-responsive visual sensors [10,11]. By fabricating structurally colored SF films—such as inverse opals or patterned nanomatrices—researchers can effectively translate the macroscopic volume expansion induced by water absorption into measurable shifts in the photonic bandgap, resulting in distinct structural color changes [12]. Recent studies have successfully demonstrated the precise design of their structural colors, revealing the capability to reconfigure and modulate these optical properties through water vapor-induced changes in protein conformation [13,14].

Despite the advancements in PC-based sensors, such as the incorporation of SF to enhance certain properties, a critical scientific challenge remains unresolved: the inherent trade-off between structural stability and high optical sensitivity in complex multiphase environments. Most structural color sensors are restricted to gaseous humidity detection, as direct exposure to liquid organic solvents or solid matrices often leads to structural collapse or severe signal distortion. Addressing this bottleneck requires a novel matrix design.

By integrating silk fibroin (SF) with highly sensitive and flexible cellulose derivatives to construct a three-dimensional inverse-opal optical architecture, we successfully engineered a bio-based photonic crystal featuring both exceptional chemical stability and high sensitivity. This advancement overcomes the limitation of conventional sensors confined solely to atmospheric humidity monitoring, extending its capabilities to the detection of trace water and the specific identification of solvents in complex liquid and solid environments. Such composite designs hold great potential for addressing the current limitations and realizing versatile, multiphase moisture detection with unprecedented visual sensitivity.

## 2. Experiments

### 2.1. Preparation of Photonic Crystal Templates

Ultrapure water was added to a four-necked flask, which was mechanically stirred and thoroughly degassed. The system was heated to 75 °C, and methyl methacrylate (MMA; polymerization inhibitor removed) was introduced. The mixture was heated further to stabilize at 80 °C, held for 15 min, then mixed with aqueous potassium persulfate (KPS). The reaction proceeded at 80 °C for 45 min. After cooling slightly, colloidal microspheres were isolated via centrifugation, washed thrice with pure water, and dispersed in pure water for shaking prior to use. Particle size was controlled by adjusting reagent quantities during synthesis.

Three-dimensional photonic crystal arrays were fabricated via the vertical sedimentation self-assembly method. A PMMA colloidal microsphere dispersion (with uniform diameter) was diluted with deionized water to 0.1–0.2 wt%, followed by 20 min sonication for homogenization. Fresh glass slides underwent hydrophilic pretreatment on both sides using a plasma cleaner, then were suspended vertically in a Petri dish. The setup was placed in a constant temperature and humidity chamber (30 °C, 40% RH), and the diluted dispersion was poured to cover the slides. After 5–7 days, complete water evaporation yielded the self-assembled three-dimensional photonic crystal arrays on the glass slides.

### 2.2. Extraction and Infiltration of Silk Fibroin

Silkworm cocoons (pupae removed) were cut into pieces, boiled in 5 g/L aqueous sodium carbonate solution for 30 min, and rinsed with pure water; this step was repeated three times to fully remove sericin protein. The degummed silk protein fibers were dried at room temperature until moisture evaporated. A 9.5 M aqueous LiBr solution (exothermic dissolution; cooled slightly after complete dissolution) was prepared, heated to 60 °C in an oil bath, and magnetically stirred. Processed silk protein fibers were added in batches and stirred at 60 °C for 4 h until completely dissolved to obtain a solution. After cooling to room temperature, the solution was dialyzed against pure water at 4 °C for 3 days using an 8000–14,000 Da dialysis bag to remove salt ions, yielding a pure silk fibroin solution.

Silk fibroin concentration: Silk fibroin solution (initial concentration ~3%) was concentrated via reverse dialysis. A 10 wt% polyethylene glycol (PEG, average molecular weight 20,000) solution was prepared as a hygroscopic agent. The solution was loaded into an 8000–14,000 Da dialysis bag, reverse-dialyzed against the PEG solution at 4 °C for 12 h to increase the concentration to ~5 wt%, and stored refrigerated for later use.

### 2.3. Humidity Examination

Determination of Relative Humidity (RH) in Air: Cut the inverse-opal-structured silk fibroin–cellulose photonic crystal film into small pieces of 1 × 1 cm and place them in the black box of the fiber-optic spectrometer, ensuring that both the fiber probe and the humidity probe are positioned above and close to the film. Gradually increase the humidity inside the sealed container using a humidifier, controlling the humidity change intervals at 5%. Once the humidity inside the box stabilizes, record the readings from the hygrometer and the reflection spectrum data. The ambient humidity within a sealed chamber containing the sample film and the fiber-optic probe was gradually increased using a humidifier. Meanwhile, the humidity levels were recorded in real time using an electronic hygrometer.

Detection of Trace Water in Organic Solvents: Acetone and isobutyraldehyde solutions with water contents of 2%, 0.25%, and 0.025% were prepared respectively. The blue-colored inverse-opal silk fibroin/cellulose photonic crystal film was immersed in the above acetone and isobutyraldehyde solutions. After standing for five minutes to allow the material to fully interact with the trace water in the solvent, the inverse-opal material was removed, and the organic solvents were allowed to evaporate. The changes in its reflection spectrum and structural color were observed and recorded.

Detection of Alcoholic Organic Solvents: The structural color of green inverse-opal-structured silk fibroin/cellulose photonic crystal material was used to detect five types of alcoholic organic solvents: methanol, ethanol, isopropanol, n-propanol, and decanol. The film was cut into 1 × 1 cm square pieces and placed on a clean glass slide. A small amount of the aforementioned organic alcoholic solvents was then dropped onto the surface of the inverse-opal photonic crystal material, ensuring complete wetting of the surface by the organic solvent. The changes in reflection spectra upon the addition of different organic alcoholic solvents were recorded using a fiber-optic spectrometer.

**Figure 1:**
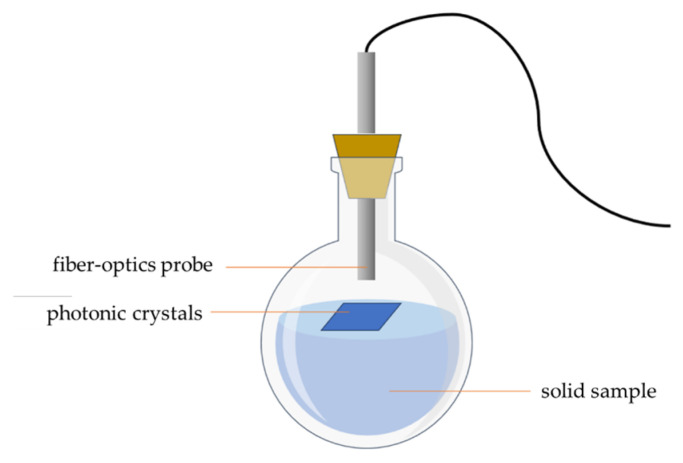
Apparatus for the determination of solid moisture content.

Detection of the Crystalline Water in Solids (Figure 1): Spray a certain mass of water onto 250 g of completely dry rice, let it stand until the rice fully absorbs the water, and prepare rice with moisture contents of 5%, 10%, and 15%. Add the treated rice with moisture to the test bottle, heat it in an 80 °C water bath to make the moisture in the rice to be tested evaporate, and record the data of the reflection spectrum changing over time. Keep the test bottle device sealed during the test process.

Spray a certain amount of water into 53 g of anhydrous sodium carbonate, so that the molar ratio of sodium carbonate to water is 1:1, 1:3 and, 1:5 respectively. After stirring evenly, seal and let it stand overnight to allow the complete reaction between sodium carbonate and water. Add the treated sodium carbonate to the test bottle, and heat it in a water bath (80 °C) to evaporate the moisture in the tested rice. Record the data of the changes in the reflected light spectrum over time. During the test, keep the test bottle device sealed.

### 2.4. Characterization of Structural and Optical Performance

At a fixed viewing angle, we recorded the visually observable structural colors of silk fibroin/cellulose photonic crystal materials, including opal and inverse-opal architectures, using a smartphone camera. We employed a fiber-optic spectrometer (Avantes Avaspec-2048TEC, Beijing, China) to determine the photonic band gaps of the three-dimensional photonic crystal array templates and the silk fibroin/cellulose composite opal and inverse-opal photonic crystals. We used field-emission scanning electron microscopy (FE-SEM) (Zeiss GeminiSEM 360, Freistaat Thuringen, Germany) to characterize PMMA colloidal microspheres of varying diameters and the morphology of the fabricated three-dimensional photonic crystal array templates, verifying the monodispersity of particle sizes and the periodic arrangement of the microspheres. We also performed FE-SEM characterization of the silk fibroin/cellulose photonic crystal composites with opal and inverse-opal structures to assess the periodicity of the photonic lattice and the structural integrity.

## 3. Results and Discussion

### 3.1. Characterization of Pmma Templates and SF/MC Composite Films

PMMA microspheres were prepared by an anionic emulsion polymerization method. In this process, only monomers and initiators were added without the use of surfactants. The size of the colloidal microspheres could be controlled by adjusting the contents of the added monomers and initiators. The dosage of the drug and the reaction conditions are shown in Table S1.

The scanning electron microscope images of four different particle sizes of PMMA nano-colloidal microspheres are shown in Figure S1. It can be seen that the prepared PMMA microspheres present a regular spherical morphology, and the particle sizes of the microspheres are uniform and highly monodisperse. Combining Table S1 and SEM Figure S1, it can be known that under the condition that other reaction conditions remain consistent (including the amount of initiator), as the amount of MMA increases, the particle size of the PMMA microspheres gradually increases. This is because the increase in the amount of monomers leads to the growth of polymer chains during the polymerization reaction being longer, thereby forming larger microspheres.

**Figure 2:**
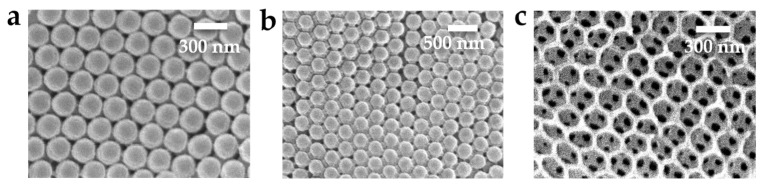
Scanning electron microscope images of (**a**) PMMA three-dimensional photonic crystal array. (**b**) Silk fibroin/cellulose photonic crystal opal structure. (**c**) Silk fibroin/cellulose inverse-opal structure.

Figure 2a shows the scanning electron microscope image of the three-dimensional photonic crystal template array prepared by the vertical self-assembly method. The overall array shows the standard face-centered cubic close packing (FCC) structure. As can be seen from the figure, the three-dimensional photonic crystal obtained by self-assembly has PMMA microspheres arranged closely and orderly, with uniform microsphere spacing, and exhibits good periodicity.

Figure 2b,c are the scanning electron microscope images of the silkworm protein cellulose proteinite structure and the inverse-opal structure. From Figure 2b, it can be seen that in the silkworm protein cellulose photonic crystal proteinite material, the structure of the PMMA array template has not been damaged, the arrangement of the microspheres still maintains a high degree of periodicity and orderliness, and the gaps between the PMMA microspheres are completely filled by the silkworm protein cellulose. As shown in Figure 2c, toluene completely dissolves the PMMA colloidal microspheres during the chemical etching process, yielding a residue-free inverse-opal material. Furthermore, this chemical etching method does not damage the overall structure of the silk fibroin/cellulose. The obtained inverse-opal structure exhibits a large-area ordered periodic porous network. However, due to a certain degree of shrinkage during the etching process, minor deformation defects can be observed in localized regions of the structure which do not affect the optical response. The inverse-opal photonic crystal obtained by etching has periodic air holes, and these air holes replace the original PMMA microspheres. The diameter of the holes replacing the PMMA colloidal microspheres is approximately 210 nm, which is about 18 nm smaller than the particle size of the microspheres. There are two factors causing this contraction: one is that the silkworm protein cellulose membrane naturally contracts after losing the support of the hard microspheres; the other is that when the film is immersed in toluene during the chemical etching process, the overall shrinkage of the film occurs during the evaporation process of toluene after the etching is completed.

**Figure 3:**
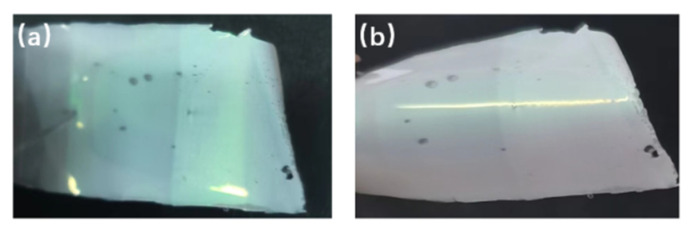
The physical images of silk fibroin/cellulose photonic crystals (**a**) placed flat, (**b**) bent.

Due to the addition of methylcellulose, the mechanical properties of the photonic crystal were improved. The silk fibroin/cellulose photonic crystal material has good flexibility, enabling the material to effectively disperse stress under external force. Therefore, it can be bent without breaking, as shown in Figure 3.

**Figure 4:**
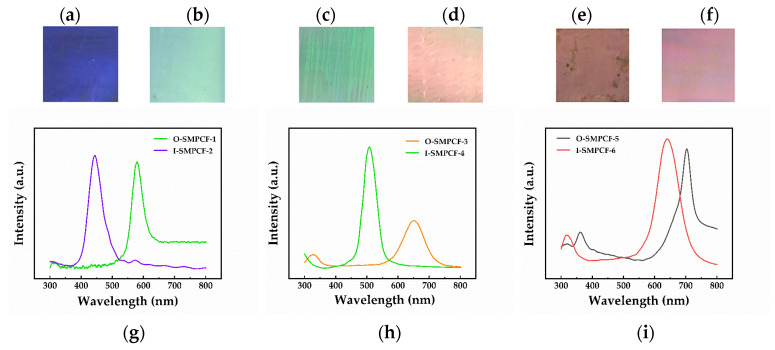
Opal structure O-SMPCF with particle size of (**a**) 228 nm, (**c**) 245 nm, (**e**) 282 nm. Corresponding inverse-opal structure I-SMPCF with particle size of (**b**) 228 nm, (**d**) 245 nm, (**f**) 282 nm. Corresponding reflection spectrum of photonic crystals with particle size of (**g**) 228 nm, (**h**) 245 nm, (**i**) 282 nm.

The structural colors and the measured reflection spectra corresponding to the opal and inverse-opal structures of the three-dimensional silk fibroin/cellulose photonic crystals (“O-SMPCF” and “I-SMPCF” for short) fabricated from PMMA microspheres of different diameters are shown in Figure 4. Specifically, Figure 4a,c,e represents the opal structures of O-SMPCF fabricated from PMMA microspheres with diameters of 228 nm, 245 nm, and 282 nm, respectively. Figure 4b,d,f represents their corresponding silk fibroin/cellulose inverse-opal structures I-SMPCF, and Figure 4g–i represents their corresponding reflection spectra. As can be seen from the figures, as the diameter of the PMMA microspheres used increases, the photonic bandgap of the corresponding photonic crystals shifts towards a longer wavelength, and the corresponding reflection spectra shift towards the red. At the same time, the film’s structural color can be observed to change from green to red. After removing the microsphere template, the reflection peaks of the films all shift towards the blue. This is due to the reduction of the periodic parameters of the photonic crystals after the microsphere template is etched, and also because the effective refractive indices of PMMA microspheres and air are different. The corresponding structural colors also change accordingly before and after etching. The film after chemical etching still has a bright structural color, which also indicates that etching does not destroy the periodic structure of the photonic crystals.

The structural color of three-dimensional photonic crystals is generated due to the photon bandgap, which is similar to crystal diffraction. Its diffraction characteristics follow the Bragg diffraction law:

(1) nλ=2dcos θ

In this equation, n is the reciprocal of the average refractive index of the crystal, λ is the wavelength of the diffracted light, d is the crystal plane spacing, and θ is the angle between the incident light and the normal of the crystal surface. This equation is the traditional Bragg equation, which was derived based on the diffraction of X-rays under vacuum conditions. Due to the different diffraction media of photonic crystals, the traditional Bragg formula needs to be modified, as follows:

(2) $$\lambda =1.633\left(\frac{d}{m}\right)\left(\frac{D}{D_{0}}\right){\left({n_{eff}}^{2}-{sin}^{2}\theta \right)}^{\frac{1}{2}}$$

In the above equation, m represents the order of Bragg diffraction, and here m = 1; $n_{eff}$ is the effective refractive index of the photonic crystal; $\frac{D}{D_{0}}$ is the swelling ratio of the photonic crystal, and here $\frac{D}{D_{0}}$ = 1; θ is the incident angle, and $n_{eff}$ can be calculated by Equation:

(3) $${n_{eff}}^{2}={n_{1}}^{2}f+{n_{2}}^{2}\left(1-f\right)$$

For the face-centered cubic arrangement, it constitutes a three-dimensional photonic crystal. In the equation, the volume fraction f is 0.74. In the opal-type photonic crystal, $n_{1}$ represents the effective refractive index of the template microsphere, which is approximately 1.49 for PMMA; $n_{2}$ represents the effective refractive index of the filling material, which is approximately 1.71 for silk fibroin/cellulose. In the inverse-opal-type film, $n_{1}$ is the effective refractive index of air, which is 1.00, and $n_{2}$ is the effective refractive index of silk fibroin/cellulose, which is approximately 1.71. Taking the 228 nm PMMA microsphere as an example, substituting the above values into the formula, the theoretical reflection peak center wavelength of the opal-type structure is calculated to be 574 nm, and the theoretical reflection peak wavelength of the inverse-opal structure is 420 nm. The theoretical calculation results are basically consistent with the actual measurement results.

### 3.2. Colorimetric Response to Atmospheric Humidity

When silk fibroin comes into contact with water, it undergoes a change in its secondary protein structure, forming a β-sheet; conversely, methylcellulose dissolves upon contact with water. Therefore, a photonic crystal prepared by combining these two materials can respond to moisture and be used to detect air humidity. As the relative humidity of the air gradually increases, water molecules adsorb onto the surface of the composite material or penetrate into the interior of the opal-like porous structure. This causes the composite material to swell and deform to a certain extent. As the moisture content increases further, this deformation becomes more pronounced and may even disrupt the periodic structure of the photonic crystal. These microstructural changes alter the optical bandgap of the photonic crystal, resulting in corresponding changes in the reflection spectrum and structural color. Consequently, silk-based cellulose–opal composite materials can be applied to the detection of air humidity.

**Figure 5:**
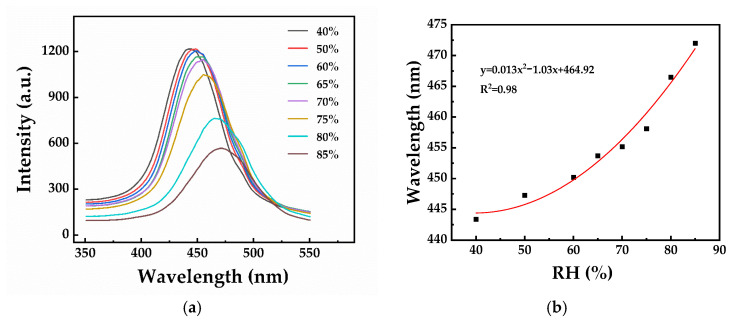
(**a**) Reflection spectra under different humidity conditions. (**b**) Relationship curve between reflection wavelength and relative humidity of silk fibroin/cellulose inverse-opal materials.

When a silk-protein-cellulose photonic crystal inverse-opal film was placed in a sealed chamber with gradually increasing humidity, the response results are shown in Figure 5. As shown in the figure, as the ambient humidity increases stepwise, the reflection peak of the film gradually red-shifts, and the intensity of the reflection peak gradually decreases. At the initial stage when the humidity is low, the red-shift of the reflection peak changes only slightly with increasing humidity; at this point, only a 10% change in relative humidity can be detected. When humidity reaches a certain value, the red-shift of the film’s reflection peak increases with rising humidity, and at this point, a 5% change in relative humidity can be detected. Furthermore, the intensity of the reflection peak also shows a more pronounced decrease at this stage. This indicates that the film is more sensitive to moisture under high-humidity conditions, and as the amount of water molecules adsorbed by the photonic crystal film increases, the periodic structure of the photonic crystal is gradually disrupted, causing the structural color to fade and the intensity of the reflection peak to decrease.

The red-shift of the photonic crystal’s reflection peak with increasing relative humidity can be attributed to two main factors. First, the polar protein chains of silk fibroin interact with the absorbed polar water molecules, inducing a conformational change in the protein structure that consequently causes the material to swell. Second, the moisture-sensitive nature of methylcellulose leads to the macroscopic swelling of the material upon water absorption. The synergistic effect of these two factors expands the lattice spacing of the photonic crystal, thereby resulting in a red-shift of the reflection wavelength.

As the humidity progressively increases, the absorbed moisture disrupts the overall architecture of the film; specifically, the periodicity of the photonic crystal is gradually compromised, leading to a continuous decrease in the reflection peak intensity. Furthermore, because this structural degradation is irreversible, the film’s response to environmental humidity is correspondingly irreversible; that is, the structure fails to recover once the ambient humidity returns to a lower level.

When the ambient humidity increases from 40% to 85%, the reflection peak of the film exhibits a red-shift of 29 nm, indicating a high sensitivity to atmospheric moisture. The sensitivity for humidity detection is 0.64 nm/%RH. Notably, the film demonstrates a more pronounced response under high-humidity conditions compared to low-humidity environments, with a minimum detectable humidity variation of 5%. It should be noted that the sensitivity is not constant but gradually increases as the humidity rises.

### 3.3. Detection of Trace Amounts of Water in Organic Solvents

The determination of water content in organic solvents is of great significance in chemical experiments. On one hand, substances that react readily with water are often stored in organic solvents. If there is a trace amount of water in the storage environment, it may cause the stored substances to undergo denaturation and damage. On the other hand, many chemical reactions have strict reaction conditions, requiring both reactants and catalysts to be absolutely free of water. If there is a trace amount of water in the system, it will cause the reactants or catalysts to denature, resulting in an increase in impurities or even the inability to proceed with the reaction. Grignard reactions are a very classic reaction in organic chemistry and are one of the most important carbon–carbon bond formation reactions. Its essence is that the Grignard reagent RMgX reacts with electrophilic reagents such as aldehydes and ketones, causing the carbon chain to grow. The Grignard reagent is an extremely strong Lewis base, which can seize protons from water or other Lewis acids. If there is water in the system, it will lead to the inactivation of the Grignard reagent and the inability to obtain the target product, so it is necessary to ensure that the system is strictly free of water when performing Grignard reactions. Determining whether there is a trace amount of water in organic solvents is very necessary for the smooth progress of organic reactions such as Grignard reactions, and it is of great significance to the pharmaceutical, food, and chemical industries. People have now developed water content detection methods such as the classic Karl Fischer method and gas chromatography, but these methods have more or less problems such as strict conditions, cumbersome experiments, expensive instruments, and the use of harmful reagents that pose health risks.

The wetting contact angle θ can measure the wetting performance of the tested liquid on the solid surface. In theory, the smaller the θ, the better the wetting property of the solvent on the solid. When θ is greater than 90°, the solid cannot be wetted by the corresponding liquid. When θ is less than 90°, the solid can be wetted. When θ is 0, the liquid completely wets the solid and spreads completely on its surface.

**Figure 6:**
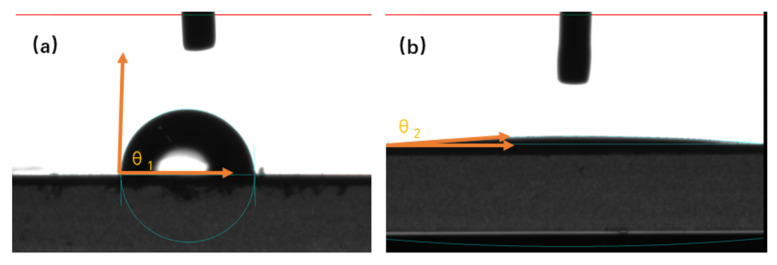
The contact angles of the silk fibroin/cellulose photonic crystal inverse-opal composite material with (**a**) water and (**b**) toluene.

Figure 6 shows the contact angles of water and the organic solvent toluene on the hydrophobic protein cellulose composite material with anti-crystalline structure. As can be seen from the figure, the contact angle of toluene on the surface of the inverse-opal composite material is 5°, indicating excellent wetting properties, while the contact angle of water on the composite material surface is 89°, which shows that its wetting properties are much lower than that of toluene. This is because when water is on the surface of the composite material, its cohesive force is stronger than its adhesion force. Therefore, water is not easy to be on the wetting film surface or penetrate into its interior, while for toluene on the material surface, the adhesion force is stronger than the cohesive force. Thus, it has good wetting properties and is easy to penetrate into the material interior. The test results of the wetting contact angle indicate that this material has strong affinity for polar solvents with low polarity, and has slightly weaker affinity for solvents with high polarity. This makes toluene and other polar organic solvents easy to penetrate into the porous structure of the inverse-opal composite membrane. Toluene and other polar organic solvents with low polarity have less damage to the inverse-opal structure of the material, while some solvents with high polarity (such as water, methanol, ethanol, etc.) will damage the inverse-opal structure. Therefore, we can use the silk fibroin/cellulose inverse-opal material and utilize its optical property changes to achieve visual naked-eye detection of trace water in organic solvents. This method is simple to operate, has low cost, and the material is non-toxic and harmless, which well overcomes the shortcomings of traditional detection methods.

**Figure 7:**
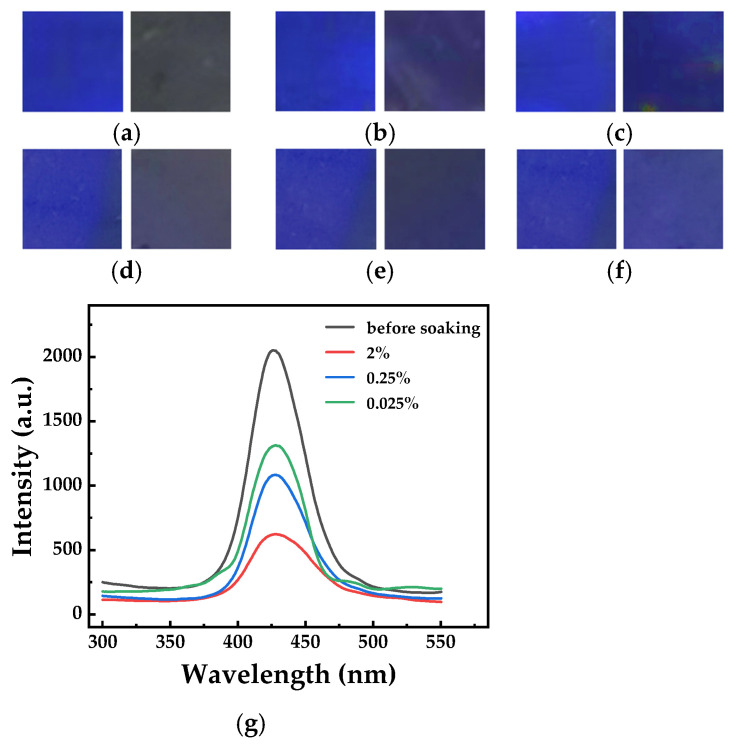
Structural color variations of the silk fibroin–cellulose inverse opals before and after immersion in acetone with water contents of (**a**) 2%, (**b**) 0.25%, and (**c**) 0.025%, and in isobutyraldehyde with water contents of (**d**) 2%, (**e**) 0.25%, and (**f**) 0.025%. (**g**) Corresponding reflectance spectra in isobutyraldehyde.

The changes in the structural color of the inverse-opal silk fibroin–cellulose films upon removal from acetone and isobutyraldehyde with varying water contents are shown in Figure 7a–e. Following immersion in the aqueous organic solvents, the structural color of the silk fibroin photonic crystals exhibited varying degrees of fading, which is attributed to the disruption of the inverse-opal structure by moisture. As the water content in the organic solvents increased, the degradation of the film’s structural color became progressively more severe. Notably, when the water content in acetone reached 2%, the structural color of the photonic crystal film vanished completely, indicating the total destruction of the periodicity within the inverse-opal structure.

The variations in the reflectance spectra of the silk fibroin–cellulose photonic crystal inverse opals upon removal from isobutyraldehyde with varying water contents are presented in Figure 7g. Use the state of the film before immersing it in the organic solvent as the blank control. As the water content in isobutyraldehyde increased, the position of the reflection peak of the film remained essentially unchanged. This indicates that while the presence of moisture affects the reflection peak intensity, it does not significantly alter the photonic bandgap position of the photonic crystals. This phenomenon may be attributed to the fact that the trace amount of water in isobutyraldehyde primarily induces irreversible structural damage to the material; however, upon the evaporation of moisture alongside the organic solvent, no significant irreversible impact is exerted on the overarching periodic structure.

With increasing water content, the intensity of the reflection peak gradually decreased, which is highly consistent with macroscopic visual observations. The attenuation of the reflection peak intensity confirms the disruption of the inverse-opal structure. Based on the degradation degree of the structural color in the silk fibroin–cellulose inverse-opal films, the water content within organic solvents can be semi-quantitatively estimated. This method offers the advantages of facile operation and low cost, while exhibiting a high sensitivity capable of detecting trace water levels as low as 0.025% in organic solvents.

By further improving the film quality to enhance its detection precision for trace water, and establishing a standard colorimetric library via precise measurements of solvent water contents, it would be highly feasible to achieve quantitative naked-eye detection of trace water in organic solvents through colorimetric analysis. Such an approach holds great promise for broad practical applications in diverse fields, encompassing chemical manufacturing, pharmaceuticals, and food processing.

### 3.4. Refractive Index-Driven Recognition of Organic Alcohols

Unlike solid opal photonic crystal structures that lack voids, inverse-opal architectures feature periodically arranged air pores. This porous configuration endows the photonic crystals with a substantially higher sensitivity to external stimuli compared to conventional opal structures. When the inverse-opal photonic crystal films are immersed in diverse organic solvents, the solvents permeate the internal structure and fill the air pores. This alteration in the surrounding medium induces a change in the effective refractive index (n_eff_). According to Equation (2), n_eff_’s variation triggers a shift in the reflection wavelength (λ), subsequently causing a change in the structural color of the photonic crystal. Such a highly porous material design bestows the inverse opals with exceptional responsiveness. Leveraging this characteristic, silk fibroin–cellulose photonic crystal inverse-opal materials can be utilized for the real-time monitoring of organic solvents.

Organic solvents exhibit excellent wettability on the silk fibroin–cellulose matrix. Consequently, upon immersion of the silk fibroin–cellulose photonic crystal inverse opals into various organic alcohol solvents, the solvents rapidly infiltrate the periodic voids within the inverse-opal structure, generating a response within a very short timeframe. According to Bragg’s law, this infiltration rapidly alters the photonic bandgap of the photonic crystals, thereby leading to distinct changes in both the reflection peak wavelength and the macroscopic structural color.

**Figure 8:**
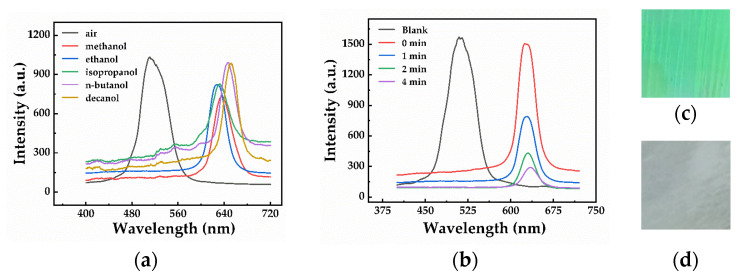
(**a**) Reflectance spectra of silk fibroin/cellulose inverse opals before and after immersion in different organic alcohols. (**b**) The time-dependent evolution of the silk fibroin/cellulose inverse opals, and structural color changes before (**c**) and after (**d**) methanol detection.

The detection results for different organic alcohols using green inverse-opal films are illustrated in Figure 8a. Following immersion in various organic alcohol solvents, the reflection peak intensities of the photonic crystal films all exhibited a slight decrease, which concomitantly resulted in a reduction in structural color saturation when observed by the naked eye (Figure 8b,c). This attenuation in reflection peak intensity is primarily attributed to two factors. On the one hand, the pristine inverse-opal voids are filled with air (*n* ≈ 1), creating a substantial refractive index contrast with the matrix material (*n* ≈ 1.71); thus, the photonic bandgap is broader, yielding a higher reflection peak intensity. Conversely, upon immersion, the voids are occupied by organic solvents (*n* > 1), which diminishes the refractive index contrast with the silk fibroin–cellulose matrix, leading to a narrowed photonic bandgap and a reduced reflection peak intensity. On the other hand, the application of solvents onto the inverse-opal forms a relatively thick liquid film on the surface. This liquid layer induces extra energy loss for both the incident and reflected light traversing it. Such supplementary energy loss decreases the reflected light intensity captured by the fiber-optic spectrometer, further attenuating the reflection peak intensity.

After immersion in different organic alcohol solutions, the maximum reflection peak positions of the inverse-opal materials all exhibited a red-shift. The effective refractive indices of methanol, ethanol, isopropanol, n-butanol, and decanol are 1.3172, 1.3611, 1.3752, 1.3993, and 1.4345, respectively. With the exception of methanol, the magnitude of the red-shift in the maximum reflection peak of the photonic crystals gradually increased as the refractive index of the organic alcohols increased. According to Equations (2) and (3), an increase in the solvent’s refractive index augments the effective refractive index of the photonic crystal, which in turn elongates the corresponding reflected wavelength; this is fundamentally consistent with the empirical measurements. The film exhibited the highest sensitivity toward decanol, reaching a maximum reflection peak red-shift of 144 nm. Owing to the highly porous structure and large specific surface area of the inverse opals, rapid mass transfer and adsorption occur upon contact with organic solvents, thereby realizing an instantaneous response. This rapid responsiveness renders the application of silk fibroin–cellulose inverse-opal films in organic solvent detection highly efficient and reliable.

Nevertheless, a critical distinction in structural stability was observed when evaluating the reversibility of the sensor. As shown in Figure 8b, while the optical responses toward higher alcohols (such as propanol, butanol, and decanol) demonstrated exceptional cycle-to-cycle reproducibility upon solvent evaporation, immersion in high-polarity lower alcohols—specifically methanol—led to a partial and irreversible degradation of the reflection peak intensity during subsequent desiccation cycles. This phenomenon is rationally attributed to the strong hydrogen-bonding capability and high polarity of methanol, which can partially disrupt the inter-macromolecular hydrogen bonds within the amorphous domains of the SF/MC matrix, inducing localized collapse or swelling of the delicate inverse-opal framework. These results underscore that while the SF/MC sensor serves as an excellent, rapid colorimetric indicator for high-boiling-point and low-polarity alcohol homologs driven by RI variations, appropriate chemical cross-linking or solvent-exposure constraints should be considered for applications involving highly aggressive polar organic solvents.

### 3.5. Evaluation of Solid Moisture and Water of Crystallization

**Figure 9:**
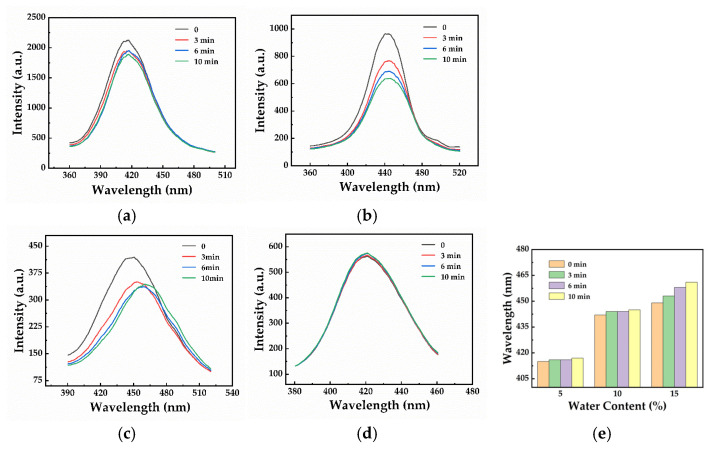
Optical responses of the silk fibroin/cellulose photonic crystal composite films to rice samples with moisture contents of (**a**) 5%, (**b**) 10%, and (**c**) 15%. (**d**) Temperature-responsive reflectance spectra of the composite films. (**e**) Variations in the maximum reflection wavelength upon detecting rice with different moisture contents.

The detection results for the moisture content in rice are presented in Figure 9. Upon heating the test vial containing the moistened rice, the elevated temperature causes the intrinsic water within the solid to evaporate into the internal atmosphere. Given that the vial is a sealed system, the relative humidity within the confined space increases. This environmental change alters the lattice parameters of the silk fibroin photonic crystals, subsequently inducing a red-shift in the reflection peak. As illustrated in Figure 9a–c, for rice with a moisture content of 5%, the reflection peak of the inverse-opal photonic crystals exhibits a gradual red-shift from an initial wavelength of 415 nm to 417 nm as the heating time increases. Similarly, for rice samples with 10% and 15% moisture contents, the reflection peaks shift from 442 nm to 445 nm, and from 449 nm to 461 nm, respectively, over the heating period. After 10 min of heating, both the wavelength and intensity of the reflection peaks stabilize, exhibiting no further variations.

To eliminate the potential interference of temperature on the detection accuracy, a control experiment was conducted. The silk fibroin/cellulose photonic crystal composite was placed in an empty test vial (in the absence of rice) and heated using an 80 °C water bath to monitor the temperature-dependent evolution of its reflection peak. As illustrated in Figure 9d, as both the heating time and temperature increased, the reflection wavelength of the film exhibited negligible red- or blue-shifts, accompanied by minimal fluctuations in peak intensity. These findings demonstrate that the detection of solid moisture content is largely unaffected by elevated temperatures, thereby confirming the reliability and feasibility of the proposed testing methodology. Variations in the maximum reflection wavelength upon detecting rice with different moisture contents were shown in Figure 9e.

Evidently, rice samples with varying moisture contents induce distinct changes in the relative humidity (RH) within the test vial. By quantitatively extracting the maximum reflection wavelengths from the measured spectra (Figure 5a) and substituting these values into the empirical humidity calibration curve (Figure 5b), the exact relative humidity (RH) inside the sealed vial can be reversely deduced. Based on this mathematical deduction, the detection of rice samples with moisture contents of 5%, 10%, and 15% corresponds to approximate RH increases of 5.8%, 8.0%, and 16.2% inside the vial, respectively. Furthermore, the observed attenuation in the reflection peak intensity suggests that the moisture evaporated partially disrupts the periodic structure of the composite film.

**Figure 10:**
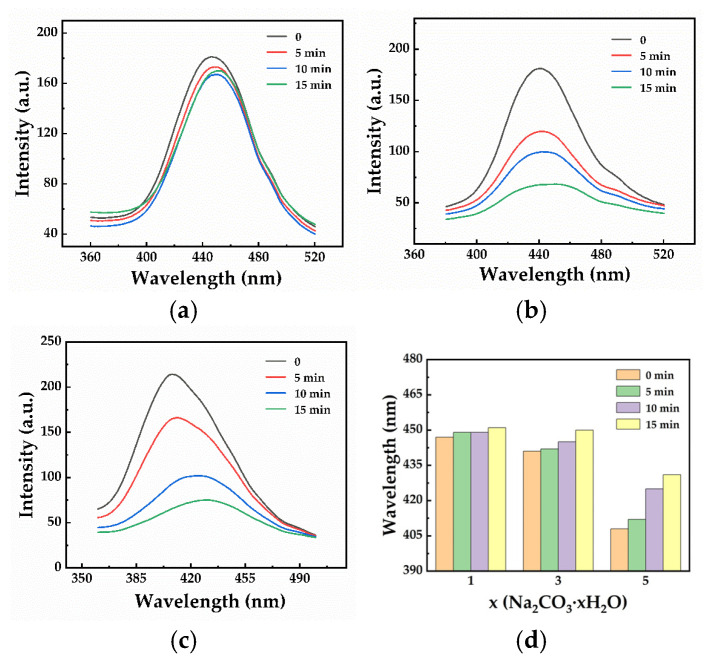
Optical responses of the silk fibroin/cellulose photonic crystal composite films to sodium carbonate with sodium carbonate-to-water molar ratios of (**a**) 1:1, (**b**) 1:3, and (**c**) 1:5. (**d**) Variations in the reflection wavelength of the composite films upon detecting sodium carbonate with varying water of crystallization contents.

The detection results for sodium carbonate containing water of crystallization are presented in Figure 10. Upon heating the test vial containing the hydrated sample, the elevated temperature induces dehydration of the solid, causing the released water molecules to evaporate into the internal atmosphere. Given the sealed nature of the vial, this leads to a consequent increase in the internal humidity. As illustrated in Figure 10a–c, for samples with sodium carbonate-to-water molar ratios of 1:1, 1:3, and 1:5, the reflection peaks of the inverse-opal photonic crystals exhibit gradual red-shifts over the heating period, shifting from initial wavelengths of 447 nm, 441 nm, and 408 nm to 451 nm, 450 nm, and 431 nm, respectively. After 15 min of heating, both the wavelength and intensity of the reflection peaks stabilize. Variations in the maximum reflection wavelength upon detecting sodium carbonate with varying water of crystallization contents were shown in Figure 10d.

As established by the prior control experiment involving the empty vial, temperature variations within the system exert negligible influence on this assay. The observed red-shift in the reflection peak directly reflects the elevated humidity within the vial, thereby verifying the presence of extractable moisture within the solid. Based on calculations derived from the fitting curve (Figure 5b), the corresponding RH increases within the vial for samples with sodium carbonate-to-water molar ratios of 1:1, 1:3, and 1:5 are approximately 8.5%, 17.9%, and 32.9%, respectively. The attenuation in the reflection peak intensity likewise indicates that the moisture disrupts the periodic structure of the film.

By further optimizing the film quality to enhance its humidity sensitivity and establishing a standard colorimetric library through precise measurements of moisture contents across various solids, it becomes feasible to achieve quantitative, naked-eye detection of solid moisture via colorimetric analysis. Particularly for the determination of water of crystallization in chemicals, this approach circumvents the need for complex and TGA. Instead, it enables efficient and convenient assessment through a facile operational procedure, thereby significantly reducing detection costs. Consequently, owing to its facile and rapid nature, the proposed composite film exhibits highly promising prospects for practical applications in the field of solid moisture detection.

## 4. Conclusions and Outlook

By integrating two eco-friendly biomaterials—silk fibroin and cellulose—a highly flexible inverse-opal photonic crystal film was fabricated to synergistically capitalize on their respective advantages. Leveraging the intrinsic water sensitivity of silk fibroin and methylcellulose, this composite film was successfully employed for moisture detection across gaseous, liquid, and solid phases. Specifically, for atmospheric humidity, an increase in relative humidity (RH) triggers a red-shift in the reflection peak of the blue film, delivering a distinct optical response even to minor RH variations of 5%. When utilized to detect trace water in organic solvents, the blue film exhibits perceptible structural color transitions at a water content as low as 0.025%. Similarly, in assessing free water and water of crystallization within solid matrices, the blue film yields a maximum red-shift of 12 nm within 10 min for moistened grains, and a 23 nm red-shift for hydrated sodium carbonate. Conversely, green films were employed for alcohol detection. Owing to the chemical stability of the composite in organic solvents, alongside the inherent porosity and solvent affinity of the inverse-opal architecture, the green film demonstrates distinct differential responses to methanol, ethanol, isopropanol, n-propanol, and decanol. Exhibiting exceptional sensitivity, particularly to decanol, this composite film holds great promise for the rapid and safe identification of diverse organic alcohols.

Despite the high performance achieved in this study, further efforts are required to bridge the gap between laboratory prototypes and real-world applications. Future research should focus on integrating this sensor into a portable, user-friendly analytical platform capable of providing direct, quantitative results for terminal users, as demonstrated in the pioneering work by Zhou et al. [15]. Such integration will involve miniaturizing the signal readout system and developing a streamlined interface, which will significantly enhance the practical utility of SF inverse opal in humidity examination.

## Data Availability

The original contributions presented in this study are included in the article. Further inquiries can be directed to the corresponding author.

## Supplementary Materials

The following supporting information can be downloaded at https://www.mdpi.com/article/10.3390/s26123875/s1, Figure S1: SEM images of different size of PMMA microspheres: (a) 191 nm, (b) 228 nm, (c) 245 nm and (d) 282 nm. Table S1. Reaction Conditions for PMMA Microsphere Synthesis.
