# Supplementary material for: Synergistic Silk Fibroin/Cellulose Inverse Opals as Flexible Colorimetric Sensors for Multiphase Water and Organic Alcohol Recognition
Source: Sensors (Basel). 2026 Jun 18;26(12):3875. doi: 10.3390/s26123875 (PMC13306377; doi:10.3390/s26123875)
Supplement: Supplementary file 1 [file sensors-26-03875-s001.zip › sensors-4353869-supplementary.pdf]

axxs

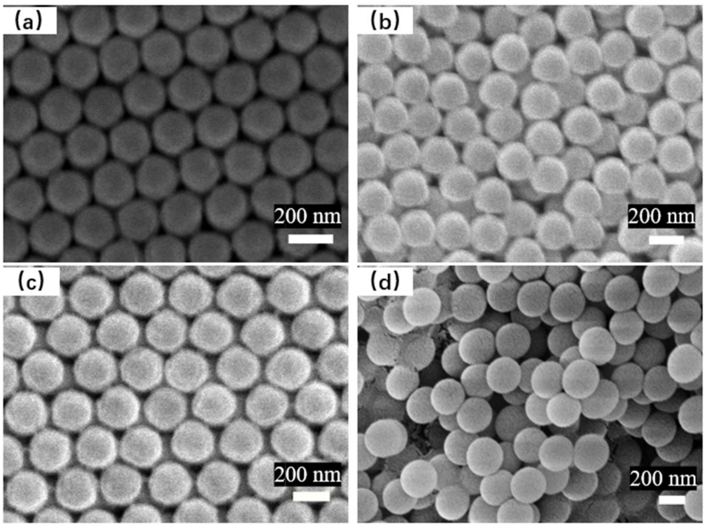

Figure S1. SEM images of different size of PMMA microspheres: (a)191 nm, (b)228 nm, (c)245 nm and (d)282 nm.

Table S1. Reaction Conditions for PMMA Microsphere Synthesis

**Entry**	MMA (mL)	Tem. (℃)	Stir (rpm)	Size (nm)
1	10	80	260	191
2	15	80	250	228
3	20	80	250	245
4	25	80	240	282

Chemicals and Materials. Methyl methacrylate (MMA, AR grade) and basic aluminum oxide (200–300 mesh) were purchased from J&K Scientific Co., Ltd. Prior to use, MMA was passed through a column packed with basic aluminum oxide to remove the inhibitor. Potassium persulfate (KPS, AR grade), sodium carbonate (Na_2CO_3, AR grade), and lithium bromide (LiBr, AR grade) were obtained from Sinopharm Chemical Reagent Co., Ltd. Polyethylene glycol (PEG, AR grade) and methyl cellulose (AR grade) were supplied by J&K Scientific Co., Ltd. Toluene and methanol of analytical grade were procured from Beijing Chemical Works. Silkworm cocoons were harvested from the Jiangnan Sericulture Base. Deionized (DI) ultrapure water used throughout all experiments was produced using an ultrapure water system (AIC-water). All commercially available chemical reagents were used as received without further purification unless otherwise specified.

Instruments and Characterization. The synthesis and processing of the materials were conducted using specialized laboratory equipment. Thermal drying was performed in a Yamato DVS402 drying oven, with the temperature regulated by a Shanghai Sile B13-3 electronic temperature controller. Solutions were agitated using a Taizhou Wanda IKA RW20 mechanical overhead stirrer or a Haimen Kylin-Medical QB-128 thermostatic shaking incubator. Ultrasonic cleaning was carried out in a Shanghai Shenyan SCQ-5201 ultrasonic

cleaner. Centrifugal separation was performed using an Anhui Jiawen JW-2019H centrifuge. Environmental conditioning was maintained within a Ningbo Haishu Saifu HWS-ISO constant temperature and humidity chamber, while low-temperature environments were managed via a Beijing Shiji Yuhua DFR-5/30 low-temperature thermostatic reaction bath. Substrate modification was executed using a Jiarun Wanfeng PC-6S plasma cleaner. Microstructures and morphology features were examined by a scanning electron microscope (Zeiss GeminiSEM 360) equipped with an energy dispersive X-ray Spectroscopy. The optical performance was investigated using an Avantes Avaspec-2048TEC fiber-optic spectrometer.
